# Reporting quality of systematic reviews with moxibustion

**DOI:** 10.1186/s13020-020-00385-z

**Published:** 2020-09-29

**Authors:** Ran Tian, Xuan Zhang, Si-Yao Li, Qi-Ying Aixinjueluo, Wai Ching Lam, Zhao-Xiang Bian

**Affiliations:** 1 Chinese Clinical Trial Registry (Hong Kong), Hong Kong Chinese Medicine Clinical Study Centre, School of Chinese Medicine, Hong Kong Baptist University, Hong Kong, SAR China; 2 China EQUATOR Centre, Hong Kong Baptist University, Hong Kong, SAR China

**Keywords:** Systematic review, Meta-analysis, Moxibustion, PRISMA statement, Reporting quality, Chinese medicine

## Abstract

**Background:**

Moxibustion is one of the major interventions of Chinese medicine (CM). The systematic reviews (SRs) are essential references for evaluating the efficacy and safety of moxibustion interventions. This study aimed to assess the reporting quality of these SRs, particularly whether necessary information related to moxibustion was adequately reported.

**Methods:**

Seven databases (including four English and three Chinese databases) were systematically searched for SRs of moxibustion that were published up to 31 December 2019. The primary analysis was to assess their reporting quality based on 27-item of the Preferred Reporting Items for SRs and Meta-Analyses (PRISMA) and 14-item of moxibustion-related information designed according to CM theory and the STandards for Reporting Interventions in Clinical Trials Of Moxibustion (STRICTOM). Descriptive statistics were also used to analyze their baseline characteristics.

**Results:**

A total of 97 SRs of moxibustion were identified from 2011 to 2019. For 27-item of PRISMA, except item 5, 8, 16 and 23, the remaining 23 items had the reporting compliances higher than 55%, of which 2 items (item 20 and 26) were fully reporting (100%). However, for moxibustion-related information, 69.1% (67/97) SRs did not provide the specific type of moxibustion, 39.2% (38/97) lacked details regarding the materials, procedure and technique used for moxibustion, 67.0% (65/97) did not report the selection criteria of acupoints for moxibustion, 28.9% (28/97) did not provide the number or duration of treatment sessions, 69.1% (67/97) did not provide any information about safety evaluation, and 94.8% (92/97) SRs did not report the treatment environment. For 51 (55.4%) of 92 SRs that included meta-analysis, it was impossible to assess whether meta-analysis had been properly conducted due to inadequate reporting of moxibustion interventions.

**Conclusion:**

The reporting quality of SRs of moxibustion need further improvements in terms of adequate reporting of moxibustion interventions and of moxibustion-related rationales. Reporting guideline of “PRISMA extension for moxibustion interventions” should be developed thus to improve their quality.

## Background

Systematic reviews (SR) can help practitioners to keep abreast of the medical literature by summarizing large bodies of evidence and explaining differences among studies on the same question. SRs can be used to inform medical decisions and to establish clinical policy. A meta-analysis (MA) is a type of SR that uses statistical methods to combine and summarize the results of several primary studies [1]. SR/MA can help clinicians to keep up to date with their field and help policymakers to judge the risks and benefits of health care behaviors. They provide a starting point for clinical practice guideline developers and summaries for funders seeking new research to support [2]. But inadequate reporting occurs in some field, such as surgery and traditional medicine etc. [3–5]. The suboptimal reporting quality of SR/MA led to the development of the Quality Of Reporting Of Meta analyses (QUOROM) Statement and its updated revision named Preferred Reporting Items for SRs and Meta-Analyses (PRISMA), published in 1999 and 2009, respectively [6, 7]. The PRISMA Statement consists of a 27-item checklist and a four-phase flow diagram, with an explanation and elaboration for each checklist item also published in 2009 [8]. The PRISMA checklists are used to guide authors of SR/MA to improve reporting quality. It is also a universal standard to assess the reporting quality of available SR/MA publications [9–11].

With a history of over 2000 years, the CM practice offers natural, safe and effective therapies and cures for many diseases with much less side effects. CM takes a unique theoretical and practical approach to health. For treatments, it includes the use of various interventions, such as moxibustion and acupuncture. The moxibustion technique is commonly used in clinical practice, including direct moxibustion with a traditional moxa stick, and indirect moxibustion. Mugwort leaves (Artemisia vulgaris, moxa) are the most commonly material used for moxibustion. Other Chinese herbal medicines could also be used in combination with mugwort. Mugwort is considered to be warm, acidic and bitter. It can promote better circulation of qi and blood by warming meridians. According to CM theory, the meridians are the channels inside the human body that circulate vital energy (in Chinese called *qi and blood*). Although it includes adverse effects, such as allergic reactions, burns and infections, sometimes, the moxibustion intervention is always believed as a safety therapy [12, 13].

As an increasing number of randomized controlled trials (RCTs) and SRs of moxibustion published in recent years, the importance to improve reporting quality of moxibustion studies has been highlighted by both researchers and users of moxibustion evidence [14–16]. In 2013, our working group has published The STandards for Reporting Interventions in Clinical Trials Of Moxibustion (STRICTOM), which aims to improve the reporting quality of RCTs with moxibustion [17]. However, no previous study has assessed the reporting quality of moxibustion SRs. Thus, this study was designed to: (i) summarize the general characteristics of all included SRs of moxibustion, (ii) assess the reporting quality of these SRs based on the PRISMA checklist, (iii) evaluate whether necessary information related to moxibustion is adequately reported, and (iv) assess whether these SRs are properly conducted in terms of synthesis of results (e.g., meta-analysis).

## Methods

### Inclusion and exclusion criteria

This study included all SR/MAs of moxibustion published in seven databases, including Embase, Cochrane Library, MEDLINE, Allied and Complementary Medicine (AMED), CNKI, VIP and Wanfang, up to 31 December 2019. Moxibustion interventions may have been given alone or in combination with other interventions or complementary alternative medicine. There were no limitations in the participants, comparisons, and outcomes. We excluded the following SR/MAs: repeat publications, research on acupuncture or other treatments as the main intervention, protocols, and withdrawal SR/MAs.

### Search strategy

The databases were searched on 13 Feb 2020 for all SR/MAs of Moxibustion that had been published up to 31 December 2019. The search terms included “meta-analysis,” “systematic review,” “moxibustion,” “mora,” “Jiu,” “vesiculation,” “Sanfu or dog days,” “Fapao or blister,” “Suanni or Garlic”, “Baijiezi or Bai Jie Zi or White mustard seed”, “Maogen or Japan Buttercup”, “acupuncture and moxibustion”, “Hanlian or Eclipta”, “Tiannanxing or Araceae or Arisaema”, etc. were included. The detailed search strategy is given in Additional file 1: S_1_.

### Screening

The titles and abstracts of the SRs were independently screened by two researchers (SY-L and RT) based on inclusion and exclusion criteria, and the full-texts of potentially suitable articles were retrieved for further assessment. Disagreements were resolved by a third reviewer (XZ).

### Data extraction

There were three pre-designed forms for data collection: (1) General characteristics form, including publication year, information of the authors, and descriptive information of included SRs. (2) PRISMA assessment form, including 27 items of the checklist. (3) Moxibustion-related information form, which was designed according to (a) CM theory and (b) STRICTOM guideline. The details are presented in Table 1.

**Table 1:** Fourteen items for reporting assessment on Moxibustion-related information

Items	Description
1	Whether a specific name of the moxibustion was reported in the “Title”?
2	Whether the CM syndrome(s) was included in the “Title”?
3	Whether the CM relevant theory was included in the “Introduction/Background” section?
4	Whether the CM diagnostic criteria of syndrome(s) were included in the “Eligibility criteria for participants”?
5	Whether CM-related outcomes (e.g., syndrome scores) were included in the “Eligibility criteria for outcome measures”?
6	Whether the environment information of moxibustion treatment was described?
7	Whether the materials and techniques used for moxibustion were reported?
8	Whether the types of moxibustion were reported?
9	Whether the selection of acupoints and meridians for moxibustion were reported?
10	Whether the number and frequency of the moxibustion sessions were reported?
11	Whether the duration of the moxibustion sessions was reported?
12	Whether any information about the adverse effects or safety assessment of the moxibustion were reported?
13	Whether the moxibustion characteristics were considered in the subgroup analysis, sensitivity analysis or other analysis of clinical heterogeneity in “Additional analyses” section?
14	Whether the heterogeneity of moxibustion interventions, such as types and dosage, has been fully considered when doing the data synthesis, especially about the meta-analysis?

### Data analysis

Two reviewers (RT and SY-L) used the PRISMA and special-designed moxibustion-related list to independently assess the reporting quality of included SRs. Each item was given a “1” score if fully reported or “0” if incompletely reported or absent. Disagreements were resolved by discussion or consultation with a third researcher (XZ). All data were collected and recorded in Microsoft Office Excel 2016. Categorical data is presented as a number (n) and percent (%).

## Results

### Search

**Fig. 1:**
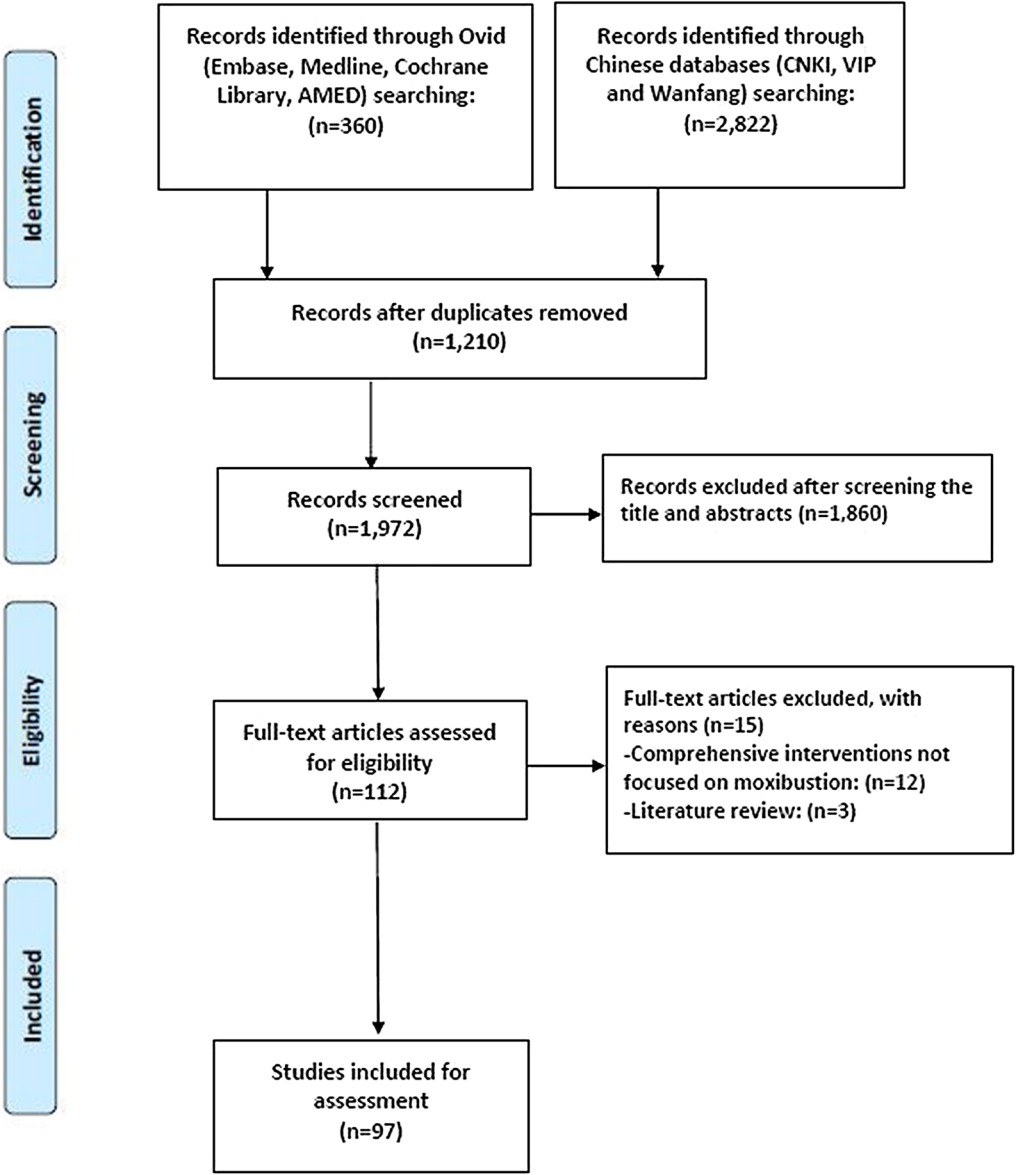
Flow chart of the performed search and selection process

Our initial literature search identified 3182 records. Preliminary screening excluded 3070 SRs due to duplication or focus on non-moxibustion interventions. After examination of the full texts of 112 articles, a total of 97 SRs was eligible for inclusion in this study (Fig. 1).

### General characteristics of included SRs

**Fig. 2:**
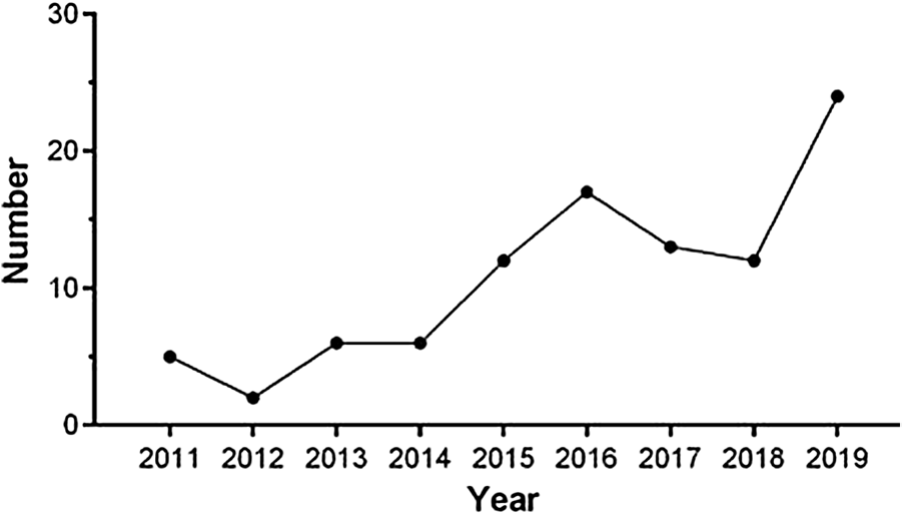
The number of included moxibustion SRs from 2011 to 2019

The first included SR of moxibustion was published in 2011, and the number of publications has been continued increasing (Fig. 2). The general features of these SRs are presented in Tables 2 and 3. The three most commonly examined conditions were diseases of musculoskeletal system or connective tissue (30.9%), diseases of the digestive system (12.4%), and diseases of the respiratory system (10.3%), respectively.

**Table 2:** General characteristics of included SRs

Category	Descriptive characteristics	N (%)
Meta-analyses	Yes	92 (94.8)
Number of authors included	1–5	64 (66.0)
	6–10	31 (32.0)
	> 10	2 (2.1)
Background of the first author	Clinician	38 (39.2)
	Researcher/methodologist	59 (60.8)
Institution of the first author	Hospital	22 (22.7)
	University	73 (75.3)
	Research Institution	2 (2.1)
Types of primary studies included	RCTs	97 (100)
Funding source	Yes	55 (56.7)
Update of a previous review	Yes	2 (2.1)

**Table 3:** Descriptive information of included SRs

Category	Descriptive characteristics	N (%)
Types of moxibustion	Natural moxibustion	11 (11.3)
	Heat-sensitive moxibustion	8 (8.2)
	Mild moxibustion	8 (8.2)
	Indirect moxibustion	3 (3.1)
	Not specific^a^	67 (69.1)
Classification of diseases^b^	Diseases of the musculoskeletal system or connective tissue	30 (30.9)
	Diseases of the digestive system	12 (12.4)
	Diseases of the respiratory system	10 (10.3)
	Diseases of the nervous system	9 (9.3)
	Diseases of the genitourinary system	7 (7.2)
	Neoplasms	5 (5.2)
	Certain infectious or parasitic diseases	4 (4.1)
	Diseases of the circulatory system	4(4.1)
	Pregnancy, childbirth or the puerperium	4 (4.1)
	Sleep–wake disorders	3 (3.1)
	Diseases of the immune system	2 (2.1)
	Endocrine, nutritional or metabolic diseases	2 (2.1)
	Mental, behavioural or neurodevelopmental disorders	2 (2.1)
	Diseases of the visual system	1 (1.0)
	Symptoms, signs or clinical findings, not elsewhere classified	1 (1.0)
	Injury, poisoning or certain other consequences of external causes	1 (1.0)

^a^The interventions were reported as moxibustion, either including all types of moxibustion interventions, or did not specify which types had been included

^b^According to International Statistical Classification of Diseases and Related Health Problems 11th Revision (ICD-11) Version

### PRISMA score of included SRs

As presented in Table 4, the total reporting rates of 27 items varied from 12.4% to 100%. Two items, including item 20 and 26, were fully reported (100%). Thirteen items, namely item 1, 2, 4, 6, 7, 9, 10, 12, 13, 14, 17, 18, and 24 were reported in more than 80% of all SRs. Eight Items, including item 3, 11, 15, 19, 21, 22, 25, and 27, were reported in more than 55% SRs. The least reported items including 5, 8, 16, and 23 were lower than 55%.

**Table 4:** Reporting quality of 27 items of PRISMA (n = 97)

Category	Item	Score, n (%)
Title	1. Title	83 (85.6)
Abstract	2. Structured summary	84 (86.6)
Introduction	3. Rationale	77 (79.4)
	4. Objective	88 (90.7)
Methods	5. Protocol and registration	12 (12.4)
Results	6. Eligibility criteria	92 (94.8)
	7. Information sources	91 (93.8)
	8. Search	49 (50.5)
	9. Study selection	83 (85.6)
	10. Data collection process	83 (85.6)
	11. Data items	69 (71.1)
	12. Risk of bias in individual studies	83 (85.6)
	13. Summary measures	82 (84.5)
	14. Synthesis of results	83 (85.6)
	15. Risk of bias across studies	63 (64.9)
	16. Additional analyses	45 (46.4)
	17. Study selection	92 (94.8)
	18. Study characteristics	94 (96.9)
	19. Risk of bias within studies	76 (78.4)
	20. Results of individual studies	97 (100)
	21. Synthesis of results	69 (71.1)
	22. Risk of bias across studies	62 (63.9)
	23. Additional analysis	31 (32.0)
Discussion	24. Summary of evidence	94 (96.9)
	25. Limitations	73 (75.3)
	26. Conclusions	97 (100)
Funding	27. Funding	55 (56.7)

### Reporting score of moxibustion-related information

As presented in Table 5, fourteen items of Moxibustion-related information were assessed. Of 97 SRs, 25.8% reported the specific name of studied moxibustion in the title, but no title included studied CM syndrome. In the included articles, few included CM syndrome-related content, such as diagnostic criteria (6.2%) and outcomes (3.1%). Relevant CM theory and rationale was provided in the Introduction among only 25.8% of included SRs. For the details of moxibustion, except one item of the materials and techniques used for moxibustion was reported in 60.8% of included SRs, the other items are seriously inadequate reporting, such as treatment environment (5.2%), the number and frequency of treatment (27.8), the safety assessment of moxibustion (30.9%), the selection of acupoints and meridians (33%), and the duration of treatment (43.3%). Besides, few SRs (27.8%) considered subgroup analysis. Based on the inadequate reporting, it is impossible to evaluate whether the synthesis of results had been appropriately conducted in more than half (55.4%) of 92 SRs with meta-analysis.

**Table 5:** Reporting quality of 14 items of Moxibustion-related information (n = 97)

Category	Item	Description	Yes, n (%)
Title	Title	1. Reported the specific name of studied moxibustion	25 (25.8)
		2. CM syndrome(s) was included	0 (0)
Introduction	Rationale	3. CM-related theory	25 (25.8)
Methods	Eligibility criteria for participants	4. Included CM syndrome diagnosis criteria	6 (6.2)
	Eligibility criteria for outcomes	5. Included CM-related outcome(s)	3 (3.1)
Results	Study characteristics	6. Reported treatment environment	5 (5.2)
		7. Reported the materials and techniques used for moxibustion	59 (60.8)
		8. Reported the types of moxibustion	30 (30.9)
		9. Reported the selection of acupoints and meridians	32 (33.0)
		10. Reported the number and frequency of the moxibustion	27 (27.8)
		11. Reported the duration of the moxibustion	42 (43.3)
		12. Reported the safety assessment or adverse effects (if exist) of the moxibustion	30 (30.9)
	Synthesis of results	13. Considered moxibustion-specific characteristics in the subgroup analysis	27 (27.8)
		14. Meta-analyses were properly conducted^a^	41 (44.6)

^a^The criteria of “properly conducted” was according to the homogeneity of the PICO information, especially the reporting quality of the details of moxibustion interventions and additional analyses provided as above. For example, if some of the moxibustion details were not reported, it is impossible to assess whether the meta-analyses in the SRs were properly conducted or not. In addition, of 97 included SRs, 92 had meta-analysis (as presented in Table 2). Thus, to calculate the proportion of this item, the percentage of records was based on the total number of 92. For example, 44.6% = 41/92

## Discussion

### General characteristics of included Moxibustion SRs

In this study, we included 97 Moxibustion SRs from 2011 to 2019 and described the baseline characteristics. Some problems have been identified. Firstly, most SRs (94.8%, 92/97) included the meta-analysis but more than half (69.1%, 67/97) did not report the specific type of studied moxibustion(s). Generally, choosing a wide range of moxibustion interventions for one disease in an SR requires rigorous methodology techniques in data analysis. Without transparent reporting, it is hard to judge the reasonable rationale for results synthesis. Secondly, very few SRs (2.1%, 2/97) have updated on time. Although it is well known that results from SRs are most useful when they are current, this study found that all included SRs published in Chinese journals did not mention any update. Based on the features of moxibustion SRs, this review is the first to systematically assess the reporting quality of moxibustion SRs with two instruments of the PRISMA checklist and moxibustion-related items. The additional moxibustion-related checklist was designed to identify the important moxibustion details in the procedure of SRs’ design, conduct, and analysis.

### PRISMA assessment of included moxibustion SRs

In this review, most SRs (84.5%, 82/97) were published in Chinese medical journals. As Chinese journals usually did not require the registration and protocol for SRs, thus the reporting of these items was very low. Consistent with previous studies, the reporting compliance of these information in Chinese SRs, particularly in the interventions of Chinese herbal medicines, acupuncture, and massage (Tuina) was seriously inadequate [18–20]. In addition, the reporting of “Search” (item 8) was unsatisfactory because more than half of the included SRs only provided some keywords for search instead of a comprehensive search strategy according to the PRISMA requirement. In comparison, the moxibustion SRs published in English journals usually have a better reporting for this item. Honestly, the inadequate reporting of “Search” could be related to the word limitation of Chinese journals, and most such journals did not provide an online appendix choice. Although most Chinese medical journal includes the endorsement of the PRISMA statement, strictly compliance and application is the key to improve the reporting of moxibustion SRs [21]. Besides, the items of “additional analysis”, either in the section of Methods or Results, were reported relatively less frequently. According to the characteristics of moxibustion, whether the additional analyses (e.g., subgroup analysis) were properly designed is the key for assessing the value of summary results [22]. Similar to previous reports, however, SRs with CM interventions (e.g., Chinese herbal medicines) usually prefer to include many different types of studied interventions [23, 24]. As the proportion of wide intervention ranges rises, and the rates of unreasonable synthesis of results more easily appeared. Thus, an appropriately pre-design of subgroup analysis is much important.

### Moxibustion-related information assessment of included SRs

For moxibustion SRs, the specific reporting items of interventions are not provided in the PRISMA checklist, so there might be a gap between the international reporting guideline and specific reporting of moxibustion SRs. Thus, we have further assessed the reporting of moxibustion-related information based on a self-designed checklist. Obviously, some deficiencies have been discovered. Firstly, we found that the inadequate and absent reporting of moxibustion details and CM related rationales are needed to be improved urgently. For SRs, whether original data items were extracted entirely and accurately is closely related to the synthesis of results. In terms of moxibustion details, the following elements are critical to be extracted: type of moxibustion, materials and technique used for moxibustion, selection of acupoints and location for moxibustion, number, frequency and duration of treatment sessions, treatment environment, and safety assessment [17]. As a result, in this study, the least frequently reported information was the number and frequency (27.8%), safety assessment or adverse effect (30.9%), the types of moxibustion (30.9%), names of acupoints and meridians (33.0%), and the duration of the treatment (43.3%). Relatively, the materials and techniques were reported in 60.8% of the included SRs. Secondly, due to the inadequate reporting of intervention details, more than half (72.2%, 70/97) SRs did not conduct subgroup analysis based on different features of moxibustion interventions (e.g., type, material, dosage). Moreover, for 92 SRs with meta-analysis, 55.4% (51/92) was impossible to assess whether data synthesis had been conducted properly. The synthesis of results includes statistical, methodological, and clinical considerations. Although the former two factors are perhaps more technical and evidence-based, the clinical considerations should be highly valued, especially for the moxibustion therapy used in clinical practice [25]. Relevant CM rationale, such as syndrome differentiation, should be proposed appropriately [26]. This content was also rarely mentioned in the included SRs, although most of them studied CM-based moxibustion.

### Improvement measures and suggestions

As some deficiencies of reporting were identified in this study, specific improvements are urgently needed. In agreement with previous findings, guidelines do help improve the quality of reporting [27, 28]. Therefore, except for continuously improved the completeness reporting of the PRISMA checklist, a series of standard reporting items especially for moxibustion SRs should be developed as an extension for the PRISMA. With the PRISMA extensions for acupuncture and Chinese herbal medicines have been published [29, 30], the specific guideline for moxibustion is helpful to supplement the SRs with CM interventions. Our group has registered this reporting guideline on the Enhancing the QUAlity and Transparency Of health Research (EQUATOR) under the item of “PRISMA for traditional Chinese medicine”, and has initiated the related work [31]. We wish to publish the Guideline soon.

### Limitations

This study has some limitations. Firstly, this review identified moxibustion SRs published up to 31 December 2019 in the targeted seven main databases. Any records which had not been included in these databases by that cut-off period have not been included. Secondly, the assessment scoring (“1” or “0”) did not allow partial information to be used. All incomplete reporting (e.g., partial and absence) were given as “0”, which decrease the reporting rate of some items. These limitations mean that the results of the study may not necessarily be comprehensive. We do however believe that the general trends indicated by the analysis of the information we did use, even if incomplete, are valid.

## Conclusions

Moxibustion SRs summarize evidence relating to efficacy and safety of moxibustion interventions—but they are valuable only if done accurately and reliably. Due to the inadequate reporting, it is a challenge to assess whether the data synthesis had been properly conducted in moxibustion SRs. Such situation seriously affects the readers’ judgments about the conclusion of these SRs and further compromises the values of moxibustion therapy. Therefore, the development of a reporting guideline for moxibustion SRs, as an extension of the PRISMA, could be an effective measure to improve the current situation.

## Supplementary information

**Additional file 1: S**_**1**_. Search strategy.

## Data Availability

The data used for this study are included in the manuscript and supplementary file.

## Abbreviations

CM: Chinese medicine
SR: Systematic review
PRISMA: Preferred Reporting Items for Systematic reviews and Meta-Analyses
STRICTOM: STandards for Reporting Interventions in Clinical Trials Of Moxibustion
MA: Meta-analysis
QUOROM: Quality Of Reporting Of Meta analyses
RCT: Randomized controlled trial
AMED: Allied and complementary medicine database
EQUAOR: Enhancing the QUAlity and Transparency Of health Research
