# Supplementary material for: Reporting quality of systematic reviews with moxibustion
Source: Chin Med. 2020 Sep 29;15:104. doi: 10.1186/s13020-020-00385-z (PMC7526112; doi:10.1186/s13020-020-00385-z)
Supplement: Supplementary file 1 — Additional file 1: S1. Search strategy. [file 13020_2020_385_MOESM1_ESM.docx]

**Supplementary information**

**Additional file 1: S_1_. Search strategy.**

Search strategy for Ovid of four English databases:

1 (((Tian or medicinal) adj3 (vesiculation or moxibustion or mora)) or Tian Jiu or Tianjiu).mp.

2 (((Sanfu or dog days) adj3 (vesiculation or moxibustion or mora)) or Sanfu#moxibustion or Sanfu or Sanfu Jiu or San Fu Jiu or Sanfujiu).mp.

3 (((Fapao or blister) adj3 (vesiculation or moxibustion or mora)) or Fapao#moxibustion or Fapao or Fapao Jiu or Fa Pao Jiu or Fapaojiu).mp.

4 ((medicine or drug or Medicine#separated) adj3 (vesiculation or moxibustion or mora)).mp.

5 ((Sanjiu adj3 (vesiculation or moxibustion or mora)) or Sanjiu#moxibustion or Sanjiu or Sanjiu Jiu or San Jiu Jiu or Sanjiujiu).mp.

6 (((Sanfu or dog day*) adj3 (sticker* or patch* or paste*)) or Sanfu or Sanfu Jiu or San Fu Jiu or Sanfujiu).mp.

7(((Baijiezi or Bai Jie Zi or White mustard seed) adj3 (vesiculation or moxibustion or mora)) or Baijiezi#moxibustion or Baijiezi or Baijiezi Jiu or Bai Jie Zi Jiu or Baijiezijiu).mp.

8 (((Maogen or Japan Buttercup) adj3 (vesiculation or moxibustion or mora)) or Maogen#moxibustion or Maogen or Maogen Jiu or Mao Gen Jiu or Maogenjiu).mp.

9 (((Banmao or Chinese Blistering Beetle) adj3 (vesiculation or moxibustion or mora)) or Banbao#moxibustion or Banmao or Banmao Jiu or Ban Mao Jiu or Banmaojiu).mp.

10 (((Hanlian or Eclipta) adj3 (vesiculation or moxibustion or mora)) or Hanlian#moxibustion or Hanlian or Hanlian Jiu or Han Lian Jiu or Hanlianjiu).mp.

11 (((Suanni or Garlic) adj3 (vesiculation or moxibustion or mora)) or Suanni#moxibustion or Suanni or Suanni Jiu or Suan Ni Jiu or Suannijiu).mp.

12 (((Tiannanxing or Araceae or Arisaema*) adj3 (vesiculation or moxibustion or mora)) or Tiannanxing#moxibustion or Tiannanxing or Tiannanxing Jiu or Tian Nan Xing Jiu).mp.

13 ((Herbal or Acupoint or acupuncture) adj3 (Patch or Applicator or Application or sticking)).mp. [mp=title, abstract, original title, name of substance word, subject heading word, keyword heading word, protocol supplementary concept word, rare disease supplementary concept word, unique identifier] {Including Limited Related Terms}

14 or/1-13

15 limit 14 to systematic reviews [Limit not valid in CDSR; records were retained]

16 limit 14 to meta analysis [Limit not valid in CDSR; records were retained]

17 15 or 16

Search strategy for CNKI:

SU=('系统综述'+'系统评价'+ 'Meta分析'+'meta 分析'+'Meta-分析'+'荟萃分析'+'元分析'+'荟萃评价'+'合并分析'+'汇总分析'+'循证评价') and SU=('灸法'+'艾灸' +'天灸'+'药物灸'+'三伏灸'+'三九灸'+'三伏贴'+'白芥子灸'+'天南星灸' +'发泡灸'+'毛茛灸'+'斑蝥灸'+'蒜泥灸'+'针灸')

Search strategy for VIP:

(M=系统综述+M=系统评价+ M=Meta分析+M=meta 分析+M=Meta-分析+M=荟萃分析+M=元分析+M= 荟萃评价+M=合并分析+M= 汇总评价+M=循证评价)* (M=灸法+M=艾灸+M=天灸+ M=药物灸+M=三伏灸+M=三九灸+M=三伏贴+M=白芥子灸+M=天南星灸+M=发泡灸+ M=毛茛灸+M=斑蝥灸+M=蒜泥灸+M=针灸)

Search strategy for Wanfang:

(“系统综述”+”系统评价”+ “Meta分析”+”meta分析”+”Meta-分析”+”荟萃分析”+”元分析”+”荟萃评价”+”合并分析”+”汇总分析”+”循证评价”) * (“灸法”+”艾灸” +”天灸”+”药物灸”+”三伏灸”+”三九灸”+”三伏贴”+”白芥子灸”+”天南星灸” +”发泡灸”+”毛茛灸”+”斑蝥灸”+”蒜泥灸”+”针灸”)
